# Electron Radiation Effects of Grain-Boundary Evolution on Polycrystalline Silicon in MEMS

**DOI:** 10.3390/mi13050743

**Published:** 2022-05-08

**Authors:** Lei Wang, Haiyun Liu, Xing Liu

**Affiliations:** 1 Key Laboratory of MEMS of the Ministry of Education, Southeast University, Nanjing 210096, China; xingliu@seu.edu.cn; 2 College of Computer and Information, Hohai University, Nanjing 211100, China; haiyun_liu@hhu.edu.cn

**Keywords:** electron irradiation, polycrystalline silicon, grain boundary evolution

## Abstract

A specimen observed with a transmission electron microscope (TEM) was processed by focused ion beam (FIB) from a surface-micromachined polycrystalline silicon MEMS structure. Electron irradiation and in situ observation were performed on a selected grain boundary in the specimen. The grain boundary was observed and located by using lattice-oriented selective TEM photography. An evolution progress of amorphization of small silicon grain within the grain boundary and recrystallization of amorphous silicon were observed. A silicon grain turned into several smaller bar grains within the grain boundary. The mechanism of grain-boundary evolution inducing a change of conductivity of polycrystalline silicon has been revealed. The conductivity of polycrystalline silicon influenced by electron irradiation could be attributed to the change of grain boundary.

## 1. Introduction

Microelectromechanical systems (MEMS) technologies are well suited for the needs of space applications due to their small size, low weight and low power consumption. One trend of space missions is to make technology smaller, faster and cheaper [1]. Different from traditional spacecraft, the shield is limited in low-weight spacecraft. Radiation from cosmic sources can potentially affect the performance of MEMS devices employed on spacecraft. Studies of the effects of radiation on MEMS devices have principally proven that there are radiation effects on the activation of mechanical elements related to charge buildup within dielectric insulating layers [2,3,4]. Displacement damage induced by radiation tends to cause resistance and stress changes in the silicon in MEMS [5,6]. The radiation effects on crystalline silicon were studied to understand the failure mechanisms of electronic devices [7,8]. There is little scientific literature on the mechanism of the radiation effects on surface-micromachined polycrystalline silicon; the radiation effects on polycrystalline silicon are much more complex than they are on crystalline silicon considering the disordered structure of grains and grain boundaries, and the field is in its infancy. The radiation effects on the resistivity of polycrystalline silicon have been studied [9,10], but the mechanism of the change of resistivity has not been revealed yet. In this article, the effects of electron irradiation on surface-micromachined polycrystalline silicon have been observed in lattice scale via a transmission electron microscope (TEM). The phenomenon of grain-boundary evolution of polycrystalline silicon has been observed, which is contributed to by amorphization and recrystallization in the grain boundaries. The change of resistance of polycrystalline silicon could be explained by this phenomenon, which may affect the potential barrier of grain boundaries due to the change of defects.

The mechanism of radiation effects on MEMS has been extended to polycrystalline materials using a grain-boundary evolution theory. This theory will help to estimate the long-term life of MEMS devices in radiation environments. One of the critical factors in the accelerated degradation experiments involving MEMS is that the failure mechanism should be the same. The observation of grain-boundary evolution in stress tests will help to determine the dose rate in accelerated degradation experiments and improve the acceleration factor. The theory will help to improve the fabrication processes of polycrystalline silicon in MEMS that are applied in radiation environments. The in situ observation of grain-boundary evolution in TEM provides a method to study the mechanism of radiation effects on different polycrystalline materials in MEMS. In addition, the further study of grain-boundary evolution will help construct the foundation of a numerical model of the radiation effects on polycrystalline silicon.

## 2. Materials and Methods

### 2.1. Fabrication of Specimen

#### 2.1.1. Surface-Micromachined Process

**Figure 1:**
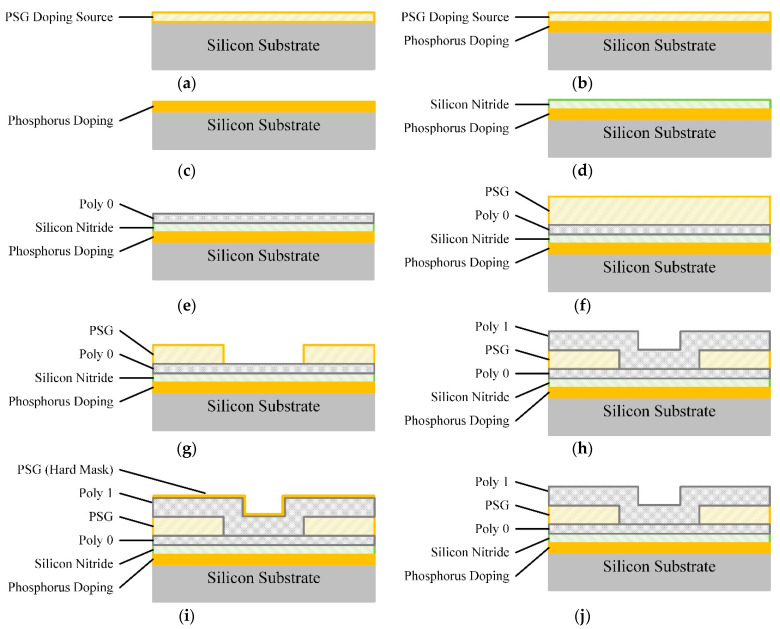
Fabrication process of specimen: (**a**) LPCVD PSG as doping source; (**b**) Doping via annealing; (**c**) Removing of PSG; (**d**) LPCVD SiN as isolation layer; (**e**) LPCVD polysilicon as underlying layer (Poly 0); (**f**) LPCVD PSG as sacrifice layer; (**g**) Etching of sacrifice layer; (**h**) LPCVD polysilicon as structure layer (Poly 1); (**i**) LPCVD PSG as doping source and hard mask of polysilicon; (**j**) Etching of polysilicon and removing of hard mask.

The specimen was fabricated in a standard MEMS fabrication process. The fabrication process began with an n-type (100) silicon wafer. The surface of the wafer was first heavily doped with phosphorus in a standard diffusion furnace using a phosphosilicate glass (PSG) sacrificial layer as the dopant source, as shown in Figure 1a,b. After removal of the PSG film by immersing the chip in a bath of 49% HF, a 600 nm low-pressure chemical vapor deposition (LPCVD) silicon nitride layer was deposited on the wafer as an electrical isolation layer, as shown in Figure 1c,d. The underlying layer of polysilicon (Poly 0) was deposited at a thickness of 500 nm, as shown in Figure 1e. A 2.0 µm PSG sacrificial layer was then deposited by LPCVD and annealed at 1050 °C for 1 h in argon, as shown in Figure 1f. The sacrificial layer was lithographically patterned with the mask in a reactive ion etch (RIE) system, as shown in Figure 1g. This step provides anchor holes that will be filled by the polysilicon layer. After etching anchor, the structural layer of polysilicon (Poly 1) was deposited at a thickness of 2.0 µm, as shown in Figure 1h. A thin (200 nm) layer of PSG was deposited over the polysilicon as hard mask, and the wafer was annealed at 1050 °C for 1 h, as shown in Figure 1i. The anneal dopes the polysilicon with phosphorus from the PSG layers both above and below it. The wafer was coated with photoresist and lithographically patterned. The PSG hard mask and polysilicon layer were etched, and the photoresist and hard mask (PSG) were removed at the end, as shown in Figure 1j.

**Figure 2:**
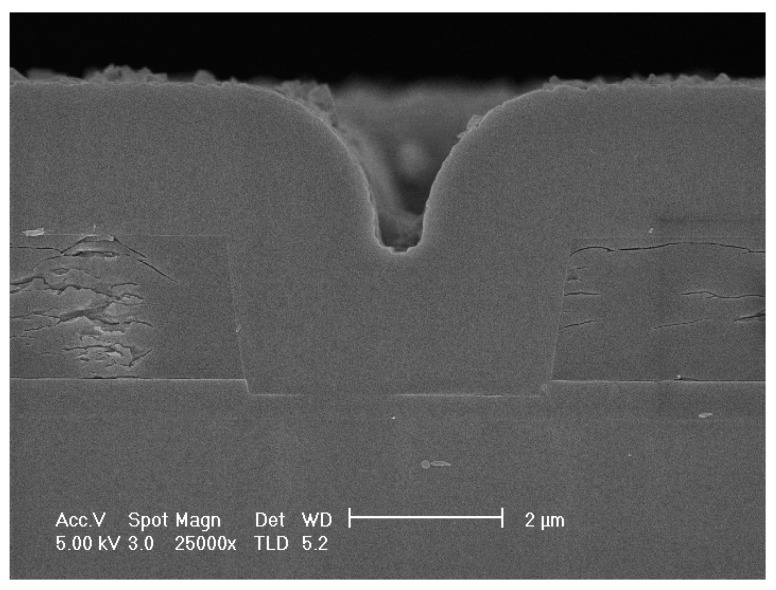
The SEM photograph of cross-section of specimen.

The scanning electron microscope (SEM) photograph of cross-section of the specimen is shown in Figure 2. The releasing process of specimen used for further experiments has not been performed. As polysilicon layer in surface-micromachined MEMS is the critical, functional part, the observation is focused on the polycrystalline silicon in irradiation environments.

#### 2.1.2. Specimen Processed by Focused Ion Beam (FIB)

The specimen observed with TEM in situ was processed by FIB from previously fabricated surface-micromachined polycrystalline silicon MEMS structure.

An 8 μm (L) × 0.1 μm (W) × 0.5 μm (T) layer of platinum was deposited by electron beam deposition on the surface of polycrystalline silicon to protect the specimen beneath. The polycrystalline silicon beam was handled by a nano-handler and sectioned by FIB, and the sectioned beam was further reduced to 0.1 μm by FIB while it was protected by the platinum layer above.

**Figure 3:**
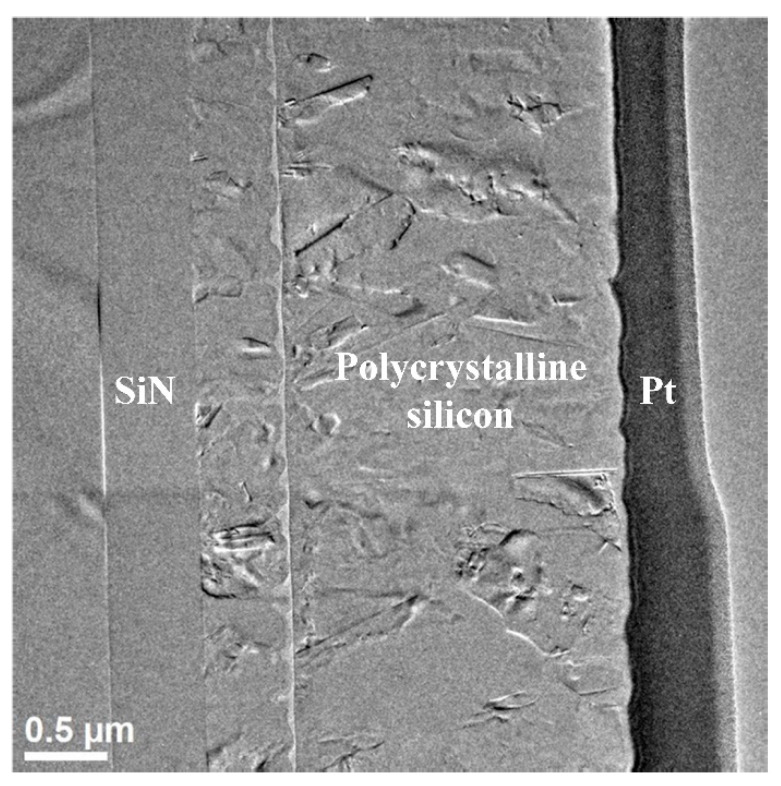
Low resolution TEM photograph of the specimen. SiN, polycrystalline silicon, and platinum can be observed.

A low-resolution figure of the FIB processed specimen observed by TEM is shown in Figure 3. At low resolution, lattice of polycrystalline silicon is not obvious, but different layers of material are shown. In this figure, from left to right, there is a substrate, one layer of SiN, two layers of polycrystalline silicon (Poly 0 and Poly 1) and one layer of platinum. The specimen is thin enough to obtain high-resolution photograph in following grain-boundary observations in TEM. A thin specimen will also help to reduce the variety of atomic arrangements in the direction of thickness and reduce the difficulty of analysis of grain-boundary evolution.

### 2.2. Experiments

#### 2.2.1. TEM Observation

**Figure 4:**
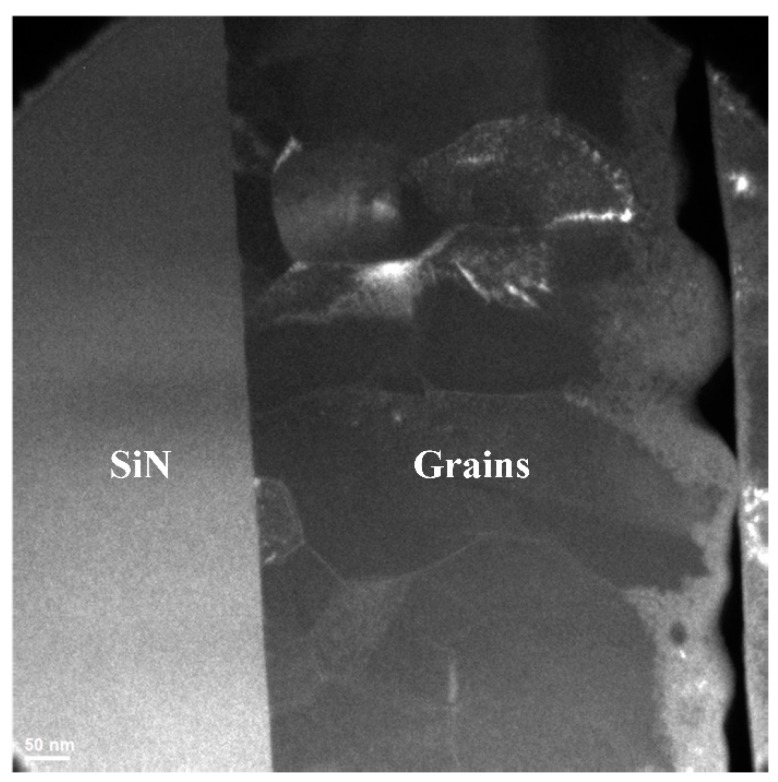
Lattice-oriented selective TEM photograph. Left part is SiN, and right part is polycrystalline silicon. Different grains have different luminances in the picture since they have different lattice orientations.

It is difficult to distinguish grains and grain boundaries of polycrystalline silicon since grains and grain boundaries are irregular in dimension. To observe and locate every grain and its boundaries, lattice-oriented selective TEM photography was performed, as shown in Figure 4. In the lattice-oriented selective TEM photograph, different grains have different luminances in the figure since they have different lattice orientations. Grain boundary is brighter since it contains some amorphous silicon. The white lines in Figure 4 show grain boundaries. The lines of grain boundaries draw the outline of every grain.

With the help of lattice-oriented selective TEM photography, grain boundaries are easily found and located for further high-resolution TEM photography. Moreover, specific grain boundary may be observed and relocated, as shown in Figure 4.

**Figure 5:**
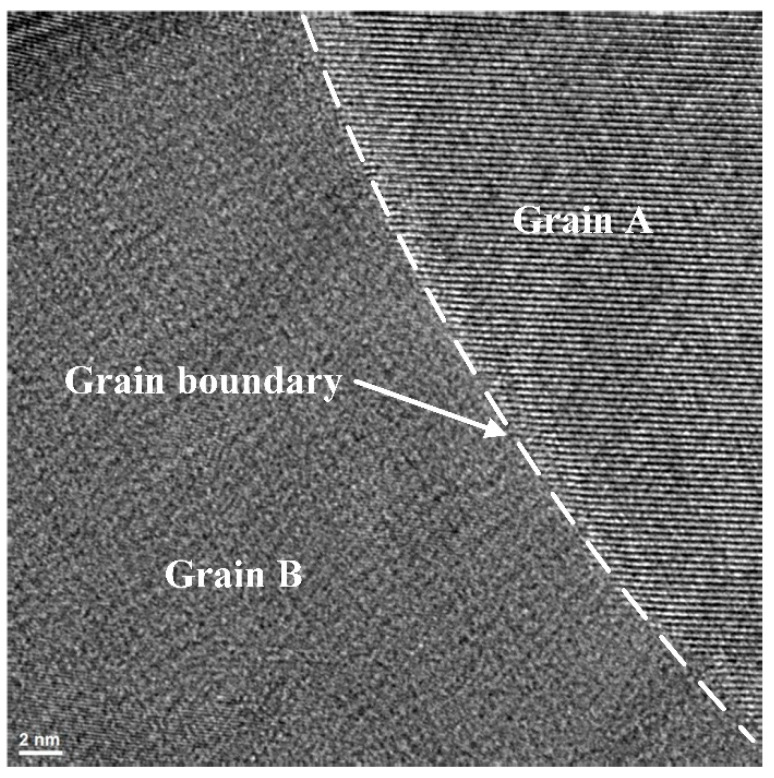
High-resolution TEM photograph of specimen. Different grains, grains’ lattice and grain boundary could be observed.

The observation point was located in a certain grain boundary, and high-resolution photography was performed. Lattice of polycrystalline silicon could be clearly observed in a narrow field of view, as shown in Figure 5. And the grain boundary between grain A and grain B could also be observed.

**Figure 6:**
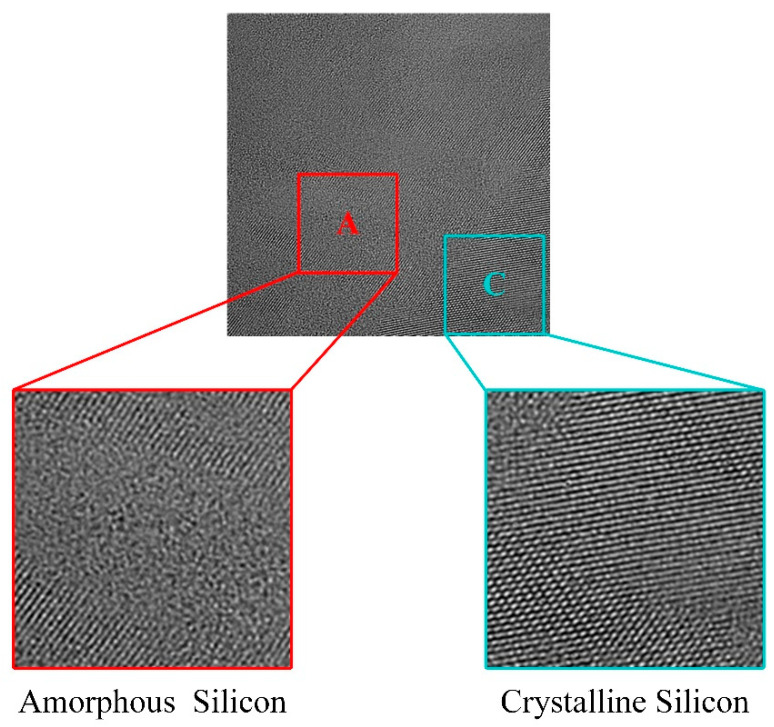
High-resolution TEM photograph in grain boundary. The zoom in area A shows the amorphous silicon part, and the zoom in area C shows the crystalline silicon part.

With the help of photography of grain boundary, the point where the followed electron irradiation and in situ observation is performed was located. The photograph before electron irradiation is shown in Figure 6. In the grain boundary, there is crystalline silicon in area A and amorphous silicon in area C. Crystalline silicon and amorphous silicon are mixed in grain boundaries.

The scale of crystalline silicon and amorphous silicon in the grain boundary ranges from several atoms to hundreds of atoms. The shape of crystalline silicon in grain boundary is usually stripe. Some of the small crystalline silicon grains in the grain boundary close to each other have the same lattice orientation. And some of the small grains have different lattice orientations.

#### 2.2.2. Electron Irradiation and Observation of Grain Boundary

A grain boundary was selected for electron irradiation and in situ observation, as shown in Figure 6. The electron irradiation was performed by using the built-in accelerated electron source in the TEM. The energy of electron beam was set at 300 keV, and the dose rate was set at 1 krad/s.

**Figure 7:**
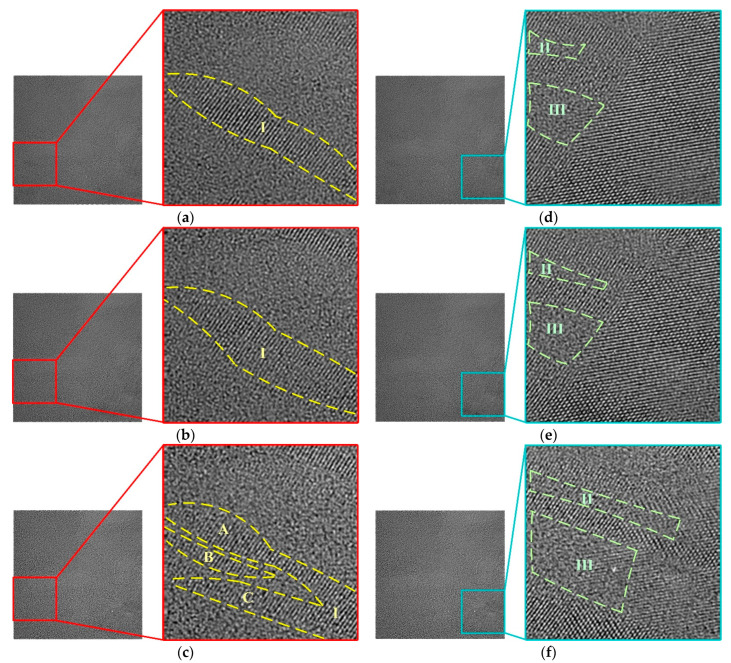
Grain-boundary evolution under electron irradiation at different doses: (**a**) zoom in area I before electron irradiation; (**b**) zoom in area I at 280 krad; (**c**) zoom in area I at 398 krad; (**d**) zoom in area II and III before electron irradiation; (**e**) zoom in area II and III at 280 krad; (**f**) zoom in area II and III at 398 krad.

Two-hundred snapshots of TEM figures were photographed in the electron irradiation experiment, and the snapshots were photographed every 2 s. Photographs at different doses were obtained from 0 to 398 krad, and the dose increment of electron irradiation was 2 krad. The grain boundary of polycrystalline silicon was observed in the snapshots, as shown in Figure 7. The amorphization of silicon grain and recrystallization of amorphous silicon were observed.

## 3. Results

The grain-boundary evolution of polycrystalline silicon was considered as the progress of amorphization of small silicon grain within the grain boundary and recrystallization of amorphous silicon.

The grain-boundary evolution is quite slow at the beginning of electron irradiation. In the first 200 s, the change of crystalline silicon and amorphous silicon in the grain boundary is small when the total dose of electron irradiation is limited to 200 krad. Between 200 s to 300 s, the crystalline silicon and amorphous silicon in the grain boundary start to change. The change of grain boundary slows down again when the time reaches 400 s.

There are two typical grain-boundary evolution mechanisms shown in Figure 7. The first mechanism is shown in area I, which is on the bottom left of the photograph. The shape of crystalline silicon in area I is a stripe before electron irradiation, as shown in Figure 7a. The crystalline silicon in area I starts the process of amorphization and recrystallization at a dose of 280 krad. The left part begins amorphization in area I, and the right part begins recrystallization in area I, as shown in Figure 7b. The shape of crystalline silicon in area I evolves from one stripe to several irregular stripes (A, B, C in area I) because of amorphization and recrystallization in area I at a dose of 398 krad, as shown in Figure 7c.

The second mechanism is shown in area II and area III, which are on the bottom right of the photograph. The shape of the amorphous silicon in area II and area III is short stripes before electron irradiation, as shown in Figure 7d. The amorphous silicon in area II and area III starts to expand at a dose of 280 krad, as shown in Figure 7e. The amorphous silicon in area II and area III expands significantly to a long stripe at a dose of 398 krad, as shown in Figure 7f.

The grain-boundary evolution is concluded as a small silicon grain turns into several smaller grains within the grain boundary, as shown in the bottom left area I in Figure 7, or the amorphous silicon expands due to the amorphization of small silicon grains within the grain boundary, as shown in the bottom right area II and area III in Figure 7.

## 4. Discussion

As shown in Figure 7a–c, the evolution in area I tends to reshape the crystalline silicon. For the trend of small silicon grain turning into several smaller grains within the grain boundary, the total volume of the crystalline silicon has changed slightly; however, the surface area of crystalline silicon increases significantly. According to the defects contained on the surface of the crystalline silicon, the defect density within the grain boundary increases with the increase in surface area. The interatomic spacing within the crystalline silicon in grain boundaries has not changed due to the lattice photography.

As shown in Figure 7d–f, the evolution in area II and III tends to increase the volume of amorphous silicon in the grain boundary. For the trend of the amorphization of small silicon grain within the grain boundary, part of the crystalline silicon is amorphized under electron irradiation. This electron radiation effect tends to increase the defect density in the grain boundary due to amorphization. The position of crystalline silicon has not changed during the amorphization of silicon grain. The density of atoms in the crystalline silicon is higher than that in amorphous silicon. The interatomic spacing in amorphous silicon tends to be closer due to the increased average atom density in the amorphous region.

The segregated phosphorus atoms in the amorphous region could not be observed in the TEM photography; the interaction between phosphorus atoms and silicon atoms needs further study.

According to the evolution of the grain boundary, two different mechanisms tend to decrease the volume of crystalline silicon within the grain boundary on average. Additionally, both mechanisms of grain-boundary evolution increase the defect density within the grain boundary.

**Figure 8:**
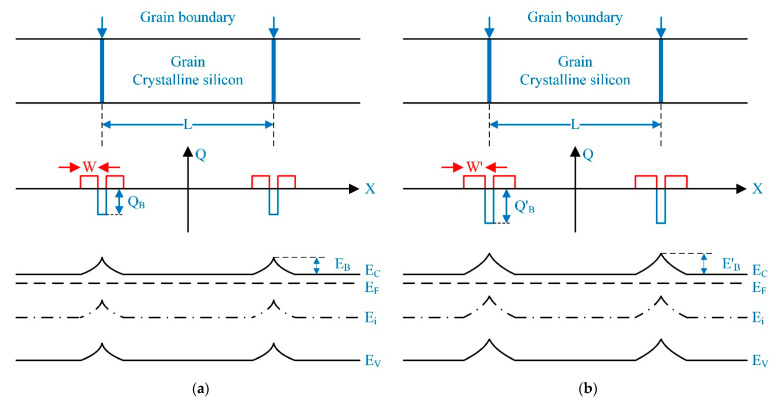
The electron radiation effect on potential barrier of grain boundary in the energy band of polycrystalline silicon: (**a**) before grain boundary evolution; (**b**) after grain boundary evolution.

The conductivity of the polycrystalline silicon is mainly contributed by a thermionic emission current other than the tunneling current due to high doping [11]. The conductivity behavior of polycrystalline silicon relies on a potential barrier induced by the trapped charge in the grain boundary, and the quantity of trapped charge depends on the defect density in the grain boundary, as shown in Figure 8a. The trapped charge in the grain boundary is considered as Q_B_, and a depletion region with a width of W in the grain is induced by the trapped charge in the grain boundary. A potential barrier with a height of E_B_ in the grain boundary is induced by the trapped charge and the charge in the depletion region.

As shown in Figure 8b, the trapped charge in the grain boundary will be increased from Q_B_ to Q′_B_ according to the increase in defect density induced by grain-boundary evolution. The depletion region in the grain will be increased from W to W′ subsequently. And the height of the potential barrier in the grain boundary will be increased from E_B_ to E′_B_.

The conductivity of polycrystalline silicon is significantly influenced by the potential barrier, since the effective mobility of carriers is figured out by the potential barrier, as shown in Equations (1) and (2) [11,12,13,14,15,16].

(1) $$\sigma =nq\mu_{eff}$$

(2) $$\mu_{\mathrm{eff}}=Lq{\left(\frac{1}{2\pi m*kT}\right)}^{\frac{1}{2}}e^{\left(-\frac{E_{B}}{kT}\right)}$$

The effective mobility of carriers will decrease according to the increase in potential barriers. The conductivity of polycrystalline silicon will decrease subsequently. The grain-boundary evolution-induced potential barrier change will decrease the mobility of polycrystalline silicon, and it will increase resistivity. This conclusion provides a theoretical mechanism for the results of research on the electron irradiation-induced resistivity change of polycrystalline silicon [9].

The increase in defect density and redistribution of defects due to the grain-boundary evolution will also affect the trap-assisted tunneling (TAT) in polycrystalline silicon [17,18]. The influence of grain-boundary evolution on TAT needs further research.

The evolution of grain boundary will influence the effective mobility of carriers in polycrystalline silicon, which is one of key factors in the conductivity mechanism of polycrystalline silicon. Most of the energy deposition of electron irradiation is in the surface of the polycrystalline silicon membrane, due to the small penetration depth of the electrons. The vertical imbalance of energy deposition should be considered when the quantitative analysis of the conductivity of polycrystalline silicon is performed.

## 5. Conclusions

Lattice-oriented selective TEM photography was performed to observe and locate the boundary for further in situ observation. Grain-boundary evolution under electron irradiation from 0 to 398 krad was observed in 200 snapshots of TEM figures. The grain-boundary evolution is concluded as a small silicon grain turns into several smaller grains, or amorphous silicon expands due to the amorphization of the small silicon grain. Both grain-boundary evolutions tend to decrease the total volume of crystalline silicon within the grain boundary and increase the defect density within the grain boundary. The trapped charge in the grain boundary will increase according to the increase in defect density, and the depletion region in the grain will increase subsequently. The height of the potential barrier will also increase. The conductivity of polycrystalline silicon will decrease due to the decrease in the effective mobility of carriers induced by the higher potential barrier. The grain-boundary evolution theory will help to explain the mechanism of radiation effects on polycrystalline silicon in MEMS. Furthermore, the temperature environment can be introduced in the TEM observation to study the radiation effects under multi-field coupling and improve understanding of the grain-boundary evolution theory.

## References

[1] Janson S., Helvajian H., Amimoto S., Smit G., Mayer D., Feuerstein S. Microtechnology for space systems. Proceedings of the 1998 IEEE Aerospace Conference.

[2] Shea H. (2009). Radiation sensitivity of microelectromechanical system devices. J. Micro/Nanolithography Mems Moems.

[3] Gorreta S., Pons-Nin J., Blokhina E., Domínguez M. (2015). A second-order delta-sigma control of dielectric charge for contactless capacitive MEMS. J. Microelectr. Syst..

[4] Dominguez-Pumar M., Gorreta S., Pons-Nin J., Gomez-Rodriguez F., Gonzalez-Castaño D.M., Muschitiello M. (2015). Closed-Loop Compensation of Dielectric Charge Induced by Ionizing Radiation. J. Microelectromech. Syst..

[5] Schanwald L.P., Schwank J.R. (1998). Radiation effects on surface micromachined comb drives and microengines. IEEE Trans. Nucl. Sci..

[6] Wang L., Tang J., Song J., Huang Q.A. Gamma irradiation effects on resistance of surface micromachined polycrystalline silicon beams in MEMS. Proceedings of the IEEE International Conference on Micro Electro Mechanical Systems (MEMS).

[7] Lin J., Wang P., Shuvra P., McNamara S., McCurdy M., Davidson J., Walsh K., Alles M., Alphenaar B. Impact of X-ray radiation on GaN/AlN MEMS structure and GaN HEMT gauge factor response. Proceedings of the IEEE 24th International Conference on Micro Electro Mechanical Systems (MEMS).

[8] Zeiler M., Nasr-Storey S., Detraz S., Kraxner A., Olantera L., Scarcella C., Sigaud C., Soos C., Troska J., Vasey F. (2017). Radiation damage in silicon photonic Mach–Zehnder modulators and photodiodes. IEEE Trans. Nucl. Sci..

[9] Wang L., Tang J., Huang Q.A. (2012). Gamma and electron beam irradiation effects on the resistance of micromachined polycrystalline silicon beams. Sensor. Actuat. A-Phys..

[10] Belwanshi V., Philip S., Topkar A. (2019). Gamma Radiation Induced Effects on the Performance of Piezoresistive Pressure Sensors Fabricated Using Different Technologies. IEEE Trans. Nucl. Sci..

[11] Seto J.Y.W. (1975). The electrical properties of polycrystalline silicon films. J. Appl. Phys..

[12] Levinson J., Shepherd F.R., Scanlon P.J., Westwood W.D., Este G., Rider M. (1982). Conductivity behavior in polycrystalline semiconductor thin film transistors. J. Appl. Phys..

[13] Lin M., Yang K.W., King Y.C. (2022). Evaluation of stability and robustness of poly-Si resistors with different dopant concentrations. Jpn. J. Appl. Phys..

[14] Narducci D., Giulio F. (2022). Recent Advances on Thermoelectric Silicon for Low-Temperature Applications. Materials.

[15] Duan L., Uddin A. (2022). Defects and stability of perovskite solar cells: A critical analysis. Mater. Chem. Front..

[16] Mizoguchi T., Imajo T., Chen J., Sekiguchi T., Suemasu T., Toko K. (2021). Composition dependent properties of p- and n-type polycrystalline group-IV alloy thin films. J. Alloy. Compd..

[17] Hogyoku M., Izumida T., Tanimoto H., Aoki N., Onoue S. (2019). Grain-boundary-limited carrier mobility in polycrystalline silicon with negative temperature dependence: Modeling carrier conduction through grain-boundary traps based on trap-assisted tunneling. Jpn. J. Appl. Phys..

[18] Hartenstein M.B., Stetson C., Nemeth W., LaSalvia V., Harvey S.P., Theingi S., Page M., Jiang C.S., Al-Jassim M.M., Young D.L. (2021). Trap-Assisted Dopant Compensation Prevents Shunting in Poly-Si Passivating Interdigitated Back Contact Silicon Solar Cells. ACS Appl. Energy Mater..

